# Hyperbaric oxygen therapy ameliorates osteonecrosis in patients by modulating inflammation and oxidative stress

**DOI:** 10.1080/14756366.2018.1485149

**Published:** 2018-10-01

**Authors:** Gerardo Bosco, Giuliano Vezzani, Simona Mrakic Sposta, Alex Rizzato, Garrett Enten, Abdullah Abou-samra, Sandro Malacrida, Silvia Quartesan, Alessandra Vezzoli, Enrico Camporesi

**Affiliations:** a Environmental Physiology Lab, Department of Biomedical Sciences, University of Padova, Padua, Italy;; b CNR Institute of Bioimaging and Molecular Physiology, Segrate (Milano), Italy;; c Department of Anesthesiology, TEAMHealth Research Institute, TGH, Tampa, FL, USA;; d Morsani College of Medicine, University of South Florida, Tampa, FL, USA

**Keywords:** Reactive oxygen species, TNF-α, IL–6, IL-1β, femoral head necrosis

## Abstract

Early stages of avascular necrosis of the femoral head (AVNFH) can be conservatively treated with hyperbaric oxygen therapy (HBOT). This study investigated how HBOT modulates inflammatory markers and reactive oxygen species (ROS) in patients with AVNFH. Twenty-three male patients were treated with two cycles of HBOT, 30 sessions each with a 30 days break between cycles. Each session consisted of 90 minutes of 100% inspired oxygen at 2.5 absolute atmospheres of pressure. Plasma levels of tumor necrosis factor alfa (TNF-α), interleukin 6 (IL-6), interleukin 1 beta (IL-1β) and ROS production were measured before treatment (T0), after 15 and 30 HBOT sessions (T1 and T2), after the 30-day break (T3), and after 60 sessions (T4). Results showed a significant reduction in TNF-α and IL-6 plasma levels over time. This decrease in inflammatory markers mirrored observed reductions in bone marrow edema and reductions in patient self-reported pain.

## Introduction

Avascular Necrosis of the femoral head (AVNFH) is a musculoskeletal condition resulting from reduced blood perfusion of the bone tissue. The natural progression of the disease often results in degenerative joint disease or complete joint dissolution^1^. Pathophysiology of AVNFH can be a result of both traumatic and atraumatic factors^2^. Currently, AVNFH is typically treated using more invasive interventions such as core decompression or total hip replacement. Ongoing studies are examining new avenues for more conservative treatments, one of which is hyperbaric oxygen therapy (HBOT). HBOT has been found to be a safe and effective therapeutic modality for management of patients with early stages of AVNFH, reducing self-reported pain scores, localized edema, and lesion size upon radiographic imaging^3^^,^^4^.

While the mechanism of action underlying the observed therapeutic effects of HBOT is not fully understood, it is known that the physio-chemistry that drives the action is a result of two factors: 100% inspired oxygen and exposure to elevated atmospheric pressure. Increased environmental pressure results in a higher number of molecules diffusing from the alveoli into the capillaries in the lungs. This coupled with 100% inspired oxygen results in a higher level of oxygen dissolved in the plasma. As a result, more oxygen is then diffused or transported to the surrounding body tissues^5^.

A proposed mechanism of action for HBOT is the resulting increase in reactive oxygen species (ROS) formation which serve as signaling molecules for multiple intracellular cascades^6–8^. A rat model developed by Asano et al. showed that HBOT elevated levels of basic fibroblast growth factor, hepatocyte growth factor, vascular endothelial growth factor, and growth response protein-1 stimulating angiogenesis and increasing blood perfusion in ischemic hind limbs^9^. Similar tissue regenerative properties were seen by Milovanova et al. who demonstrated the ability of HBOT to stimulate vasculogenic stem cell growth and differentiation^10^. The increase in these protein levels and differentiation of cells may be linked to ROS’s ability to initiate downstream changes in several transduction cascades such as stem/progenitor cell mobilisation from bone marrow and lowering monocyte chemokine synthesis, which ultimately lead to wound neovascularisation and improved post-ischemic tissue survival, respectively^8^^,^^11^.

To date, there is limited information on the molecular mechanism of HBOT action on patients with AVNFH. Key players in bone turnover include specific cytokines osteoprotegerin (OPG), receptor activator of NF-kB (RANK), and of its ligand (RANKL). Any disturbance in the OPG/RANKL/RANK equilibrium will result in a shift to either bone resorption or formation.

Previous studies have indicated interaction between inflammatory factors and the OPG/RANK/RANKL homeostasis^12^^,^^13^. In particular, the interrelations of interleukin 6 (IL-6) and interleukin 1 beta (IL-1β), RANKL, and tumor necrosis factor alfa (TNF-α), highlight the interplay in bone resorption in the pathophysiology of AVNFH^13^. The aim of our study is to investigate HBOT effect on the ROS production in AVNFH patients and its alteration of inflammatory cytokines levels.

## Material and methods

### Patient selection

The study involved 23 male patients (Table 1) with unilateral femoral head necrosis, including post-traumatic femoral head necrosis, post steroid therapy femoral head necrosis, femoral condyles necrosis and other aseptic bone necrosis. Patients with others underlying pathologies were excluded from this study. Informed consent was obtained from all patients before the start of the study. This study was approved by the Institutional Ethics Committee of the University of Padova and was conducted in accordance with the ethical standards of the Helsinki Declaration. Every patient had a plain X-ray of the hip in two projections (anterior and lateral) and then had magnetic resonance imaging (MRI) to stage their pathology according to the Ficat classification^14^ and previously published^3,4^. Ficat and Arlet proposed the original classification of avascular necrosis in 1964 before the advent of MRI; it is staged based on plain X-ray. Stage I is pre-radiologic finding, presenting with pain only, and is the earliest clinical manifestation of the syndrome. The crescent sign is a late Ficat stage II finding, a linear subcortical lucency representing a fracture line and impending femoral head collapse. Stages I–II were described as early stages and Stages III and IV were classified as late stages.

**Table 1.** Demographic features and levels of severity of disease of patients with ANFH.

Patients’ characteristics				
		Ficat stage		
Subjects (*n*)	Age (*y*)	I	II	III
23	54.2 ± 10.1	1	7	15

### Working protocol

**Figure 1.**
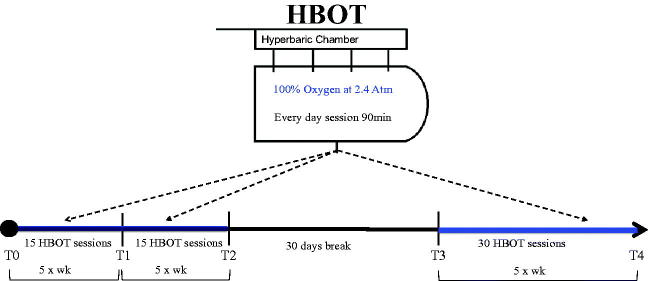
Experimental design of working protocol with timeline of blood samples collection.

As previously described^3^^,^^15^, patients were exposed to 100% inspired oxygen at 2.5 atmospheres absolute in a multiplace pressure chamber for 90 min using an overboard demand regulator and oral-nasal mask. All patients received 30 treatments of HBOT for 5 days a week for a period of 6 weeks. After a 30-day break, a second 30 HBOTs cycle was given (Figure 1).

### Blood sample collection

Blood samples were drawn from the antecubital vein. A total of 5 ml were collected in heparinized vacutainer tubes (Becton Dickinson and Company, UK). Plasma was separated by centrifuge at 1000 g for 10 min at 4 °C. Blood samples were collected at specific points during patient treatment to analyse IL-1β, IL-6 and TNF-α plasma levels as well as ROS generation as graphically represented in Figure 1. Blood was collected prior to initiation of HBOT (T0), after 15 sessions of HBOT (T1), after 30 sessions of HBOT (T2), after the 30-day break between HBOT cycles (T3), and after patient completion of HBOT (T4).

#### Quantification of plasma levels of inflammatory markers

IL-1β, IL-6 and TNF-α plasma levels were determined by ultrasensitive ELISA immunoassays (R&D Systems, Minneapolis, MN, USA), according to the manufacturer’s instructions. A nine well micro-plate was pre-coated with monoclonal antigen specific antibodies designed to target our inflammatory markers of interest (IL-1β, IL-6 or TNF-α), three plates per antigen for a positive and negative control standards to ensure there was no cross contamination. Standards and samples (∼200 µL) were pipetted into the wells and the immobilized antibody bound any antigen of interest present. Following the washing procedure, an enzyme-linked, antibody specific, polyclonal antibody was added to the wells. After subsequent washing, a substrate solution was added to the wells and color developed in proportion to the amount of cytokine bound at the initial step. The signal was then spectrophotometrically measured at a wavelength of 450 nm. Plasma levels of inflammatory markers in pg/mL were then calculated according to optical density of each well. This process was repeated for the plasma isolated from each blood sample taken and aggregated for each specific point of measure (T0, T1, T2, etc.) for comparison.

### ROS detection

A X-band electron paramagnetic resonance (EPR) instrument (E-scan-Bruker BioSpin, GmbH, MA) was used for determination of ROS. The instrument is designed to function with very low concentrations of paramagnetic species in small (50 µL) samples. For each recruited participant, the ROS production rate was determined by means of a recently implemented EPR method^16^^,^^17^. Determination involved analyse 50 µL plasma samples treated with a CMH (1-hydroxy-3-methoxycarbonyl-2,2,5,5-tetramethylpyrrolidine) probe solution (1:1), in order to transform ROS into more stable radical species that are EPR detectable. 50 µL of the obtained solution was then put in a glass EPR capillary tube (Noxygen Science Transfer & Diagnostics, Germany) that was placed inside the cavity of the E-scan spectrometer for data acquisition.

Acquisition parameters were inclusive of a microwave frequency of 9.652 GHz; modulation frequency of 86 kHz; modulation amplitude of 2.28 G; sweep width of 60 G, microwave power of 21.90 mW, number of scans was 10; and receiver gain was equivalent to 3.17·10. Sample temperature was first stabilized and then kept at 37 °C by the temperature and gas controller Bio III unit, interfaced to the spectrometer. Spectra were recorded and analysed using the Win EPR software (2.11 version) supplied by Bruker. EPR measurements allowed us to obtain a relative quantitative determination of ROS production rate in samples. All data were, in turn, converted into absolute concentration (µmol·min^−1^) by adopting CP• (3-Carboxy-2,2,5,5-tetramethyl-1-pyrrolidinyloxy) stable radical as an external reference. This process was also repeated for the plasma isolated from each blood sample taken and aggregated for each specific point of measure (T0, T1, T2, etc.) for comparison.

### Statistical analysis

Data were analysed using repeated Shapiro-Wilk-s tests. Experimental data were compared using ANOVA repeated measures with Tukey-s multiple comparison test to further check the among-groups significance. Data are presented as means ± SD All *p* values were two sided and a *p* value < .05 was considered statistically significant.

## Results

**Figure 2.**
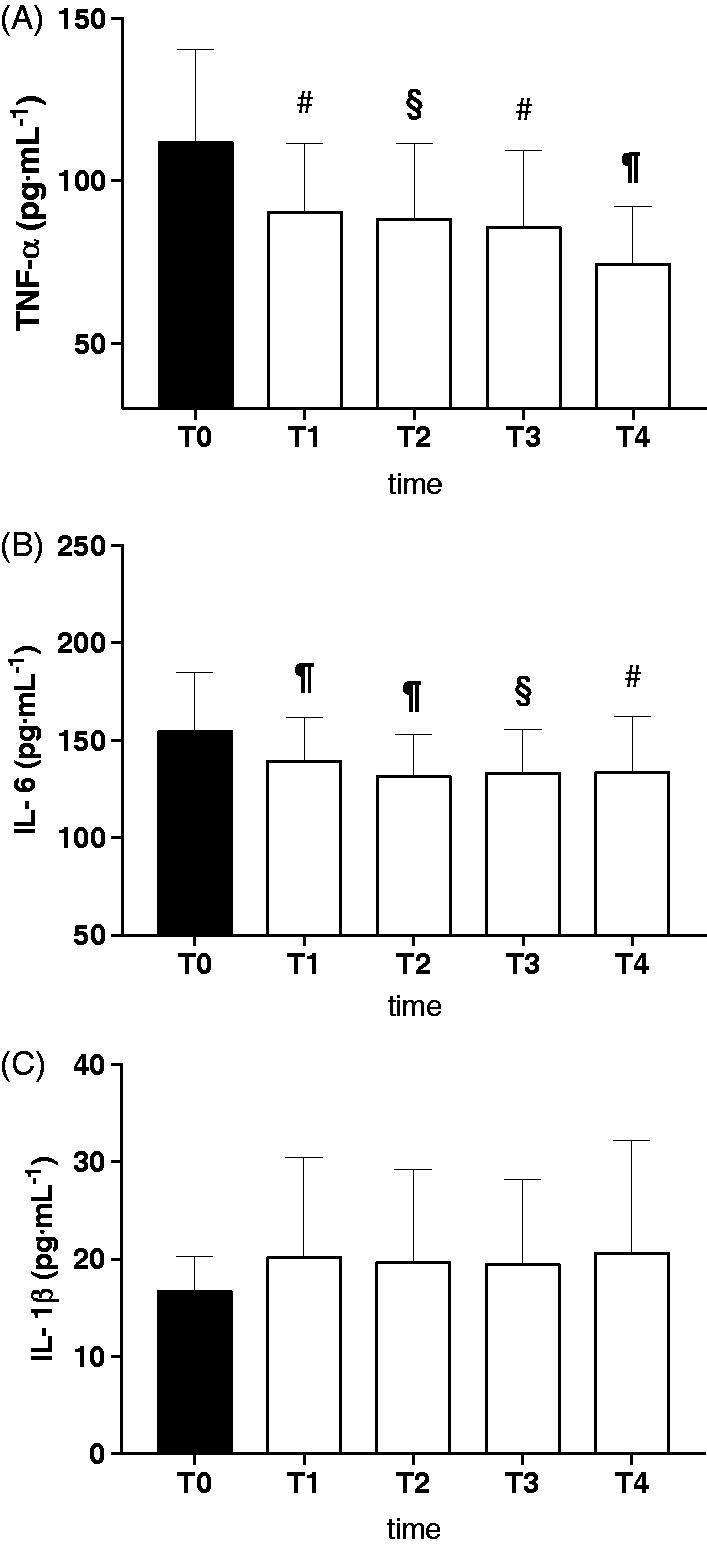
Effects of HBOT on plasma levels (pg·mL^−1^) of: (A) TNF-α, 9B) IL-6 and 9C) IL-1β in ANFH patients are shown. T0 before the beginning of the first HBOT cycle of treatments (filled bars), T1: after 15 HBOT, T2: after 30 HBOT, T3: beginning of the second HBOT cycle after a 30 days break, T4: end of the second HBOT cycle (empty bars). Data are presented as mean ± SD. Significance of differences: *P* < .05 (*), *P* < .01 (#), *P* < .001 (§), *P* < .0001 (¶).

A significant reduction in TNF-α levels from T0 (111.87 ± 28.74 pg·mL^−1^) to T1 (90.32 ± 21.26 pg·mL^−1^; *p* < .01) was observed (Figure 2(A)). Significant reductions were also seen between T0 and T2 (88.25 ± 23.32 pg·mL^−1^; *p* < .001), T3 (85.77 ± 23.72 pg·mL^−1^; *p* < .01) and T4 (74.46 ± 11.81 pg·mL^−1^; *p* < .001). Similarly, significant reductions in IL-6 levels were seen between T0 (154.47 ± 30.52 pg·mL^−1^) and T1 (139.14 ± 22.82 pg·mL^−1^; *p* < .0001), T2 (131.69 ± 21.44 pg·mL^−1^; *p* < .001), T3 (133.04 ± 22.50  pg·mL^−1^; *p* < .001), and T4 (133.38 ± 29.00 pg·mL^−1^; *p* < .001) (Figure 2(B)). No significant change in IL-1β plasma levels were seen in any subjects (Figure 2(C)). Of note, as previously described within the literature, calculated baseline plasma cytokine levels indicate an elevated inflammatory state in patients with AVNFH when compared to normal healthy individuals^18^^,^^19^.

ROS production increased from T0 (0.24 ± 0.03 µmol·min^−1^) to T1 (0.29 ± 0.05 µmol·min^−1^; *p* < .01) and T2 (0.35 ± 0.10 µmol· min^−1^; *p* < .01), however, this was followed by a gradual return to baseline values (T3: 0.30 ± 0.05 µmol·min^−1^, and T4: 0.26 ± 0.07 µmol·min^−1^).

## Discussion

In patients with AVNFH, reduced repair capacity and altered bone remodeling play an important role in progression and severity of the disease. Therefore, returning cytokines to homeostatic levels may result in treatment by modulating the interaction of IL-1β, IL-6 and/or TNF-α with OPG/RANK/RANKL.

Abu-Amer and colleagues described two isoforms of TNF-α receptors. Type 1 (p55r) was found to promote osteoclastogenesis, while Type 2 (p75r) was found to suppress osteoclastogenesis^20^^,^^21^. We hypothesize that HBOT leads to reduced levels of TNF-α decreasing binding of TNF-α to the p55r Type 1 receptor, thus decreasing the activation of NF-kB^13^. Moreover, Kurokouchi and colleagues found TNF-α to increase the expression of IL-6 genes^22^. As a result, lower levels of TNF-α resulting from HBOT exposure could explain the reduction in IL-6 levels as shown in Figure 2.

In vivo studies of IL-6 found that in transgenic mice with overexpressed IL-6 there is greater bone turnover, reduced osteoblasts, and increased osteoclasts leading to osteopenia^23^. Correspondingly, IL-6 deficient mice displayed reduced osteoclasts and lower levels of bone erosion^24^. In the inflammatory process, monocytes/macrophages produce IL-6, which can directly stimulate pre-osteoclast cells to differentiate and activate^13^. IL-6 also stimulates stromal/osteoblastic cells to produce effectors, which will then promote osteoclastic differentiation^13^.

IL-1β is one of the highly expressed and driving cytokines in inflammation. Although belonging to a structurally different cytokine class, IL-1β resembles many of the biological activity of TNF-α. Increased IL-1β levels result in downstream activation of NF-kB and c-jun N-terminal kinase which has an important role in multinucleation and bone resorption^20^^,^^25–28^. Although IL-lβ and TNF-α have been reported to show identical effects on osteoblast-mediated osteoclastic bone resorption they have contrasting effects on bone formation through osteoblastic differentiation in bone metabolism.

IL-1β enhances and TNF- α inhibits bone morphogenetic protein (BMP) -2 (osteogenin), or -4 (osteoinductive protein-l) -induced alkaline phosphatase (marker of osteoblastic differentiation) activity^29^. In the osteolytic process, TNF-α acts principally on osteoclasts precursors while IL-1β increase bone resorption indirectly through the production of RANKL whose plasma levels have been reported to be unaffected by HBOT^15^^,^^30^. However, HBOT does seem to induce upregulation of serum OPG production. This increase may reflect a compensatory response linked to the trend of IL-1β concentration as OPG secretion is induced by IL-1β^31^.

HBOT enhances healing of necrotic wounds by stimulating angiogenesis, fibroblast proliferation, osteoblast proliferation, and collagen formation. These mechanisms are stimulated by modulation of oxygen sensitive transcription factors as well as ROS-mediated signaling pathways themselves^8^. ROS levels increased between start of treatment (T0) and after 15 treatments of hyperbaric oxygen (T1) due to the hyperoxia exposition^32^. Thereafter this increase in ROS subsided after 30 treatments and was no longer witnessed in the later stages (T2, T3, T4). One possible explanation is that the first HBOT cycle exerts a preconditioning activity by upregulating the endogenous antioxidant and detoxification capacity, enhancing cellular protection against the subsequent oxidative stress damage^5–7^^,^^20^. These protective effects could be attributed to a more effective antioxidant production and activity, which occur during HBOT treatments.

The current study has potential limitations. A larger sample size, with a more homogenous clinical sample and additional pro and anti-inflammatory markers would be helpful to confirm our results. Moreover, information on transcription factors expression and specific target genes involved in cellular response to such treatments could help to better elucidate the detailed aspects of the intricate and complex link between the inflammatory, oxidative stress pathways and HBOT.

Finally, more clinical and basic researches are requested to better understand HBOT’s molecular mechanisms of action to gain evidences for this treatment to improve protocols and achieve greater resolution for patients.

## Conclusions

**Figure 3.**
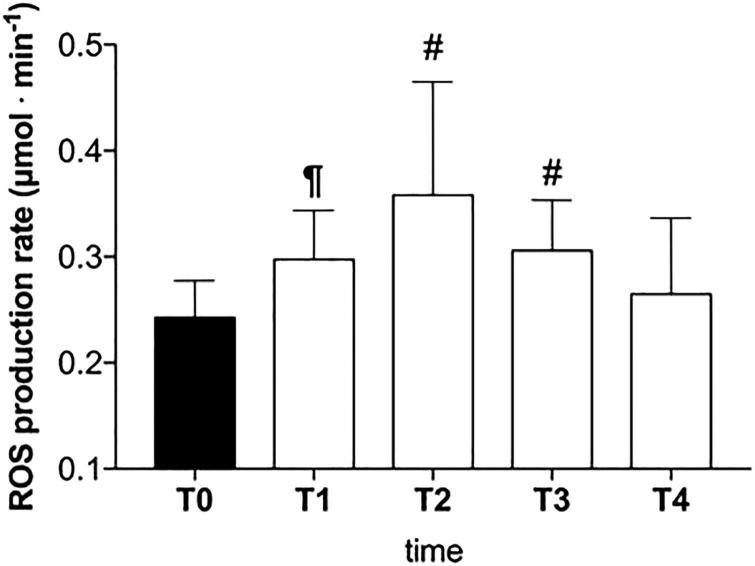
Effect of HBOT on plasma level of ROS production (μmol·min^−1^) in ANFH patients is shown. T0 before the beginning of the first HBOT cycle of treatments (filled bar), T1: after 15 HBOT, T2: after 30 HBOT, T3: beginning of the second HBOT cycle after a 30 days break, T4: end of the second HBOT cycle (empty bars). Data are presented as mean ± SD. Significance of differences: *P* < .05 (*), *P* < .01 (#), *P* < .0001 (¶).

This study shows that HBOT results in an anti-inflammatory action in patients with AVNFH. In AVNFH, HBOT results in a decreased amount of circulating TNF-α and IL-6 (Figure 2). HBOT acting on IL-1β, TNF-α, and IL-6, key bone-resorbing cytokines and their synergistic effects, could ultimately lead to beneficial resolution for the patient. Moreover the decrease in inflammatory markers mirrored the reductions in visible edema upon radiographic imaging and reductions in patient pain previously observed within the literature. The initial observed increase in ROS production enhances healing of necrotic tissues inducing antioxidant production and detoxification activity too (Figure 3).
